# Molecular Targets for Pharmacotherapy of Head and Neck Squamous Cell Carcinomas

**DOI:** 10.3390/cimb47080609

**Published:** 2025-08-01

**Authors:** Robert Sarna, Robert Kubina, Marlena Paździor-Heiske, Adrianna Halama, Patryk Chudy, Paulina Wala, Kamil Krzykawski, Ilona Nowak

**Affiliations:** 1 Silesia LabMed, Centre for Research and Implementation, Medical University of Silesia in Katowice, 40-752 Katowice, Poland; robert.sarna@sum.edu.pl (R.S.); rkubina@sum.edu.pl (R.K.); marlena.pazdzior@sum.edu.pl (M.P.-H.); paulina.wala@sum.edu.pl (P.W.);; 2 Department of Pathology, Faculty of Pharmaceutical Sciences in Sosnowiec, Medical University of Silesia, 41-200 Sosnowiec, Poland; 3 Department of Immunology and Serology, Faculty of Pharmaceutical Sciences in Sosnowiec, Medical University of Silesia in Katowice, 41-200 Sosnowiec, Poland; 4 Department of Molecular Biology, Faculty of Pharmaceutical Sciences in Sosnowiec, Medical University of Silesia, 41-200 Sosnowiec, Poland

**Keywords:** HNSCC, PD-1, PD-L2, chemotherapy, CDK, PARP, targeted therapies

## Abstract

Head and neck squamous cell carcinomas (HNSCCs) represent a heterogeneous group of tumors with a complex molecular profile. Despite therapeutic advances, patient prognosis remains poor, emphasizing the need for more effective treatment strategies. Traditional chemotherapy, with cisplatin and 5-fluorouracil (5-FU), remains the gold standard but is limited by toxicity and tumor resistance. Immunotherapy, particularly immune checkpoint inhibitors targeting programmed cell death protein 1 (PD-1) and its ligand (PD-L1), has improved overall survival, especially in patients with high PD-L1 expression. In parallel, targeted therapies such as poly (ADP-ribose) polymerase 1 (PARP1) inhibitors—which impair DNA repair and increase replication stress—have shown promising activity in HNSCC. Cyclin-dependent kinase (CDK) inhibitors are also under investigation due to their potential to correct dysregulated cell cycle control, a hallmark of HNSCC. This review aims to summarize current and emerging pharmacotherapies for HNSCC, focusing on chemotherapy, immunotherapy, and PARP and CDK inhibitors. It also discusses the evolving role of targeted therapies in improving clinical outcomes. Future research directions include combination therapies, nanotechnology-based delivery systems to enhance treatment specificity, and the development of diagnostic tools such as PARP1-targeted imaging to better guide personalized treatment approaches.

## 1. Introduction

Head and neck squamous cell carcinoma (HNSCC) is one of the most common cancers worldwide. HNSCC ranks sixth in terms of global cancer incidence and accounts for approximately 90% of all head and neck malignancies. Annually, there are over 870,000 new cases and more than 450,000 deaths attributed to HNSCC worldwide [1,2,3,4]. Epidemiological data show that approximately 75% of HNSCC cases are strongly associated with tobacco use and alcohol consumption [3]. Notably, the incidence of HNSCC varies by region and country, largely due to differing levels of exposure to these and other risk factors [2].

HNSCC typically arises from the mucosal lining and is most commonly located in the oral cavity, sinuses, pharynx, and larynx. In addition to smoking and alcohol abuse, infections with human papillomavirus (HPV) and Epstein–Barr virus (EBV) are key risk factors [1,2,4]. Given the significant role of HPV in carcinogenesis, HNSCC is often classified into HPV-positive and HPV-negative subtypes [2]. This classification was proposed because of numerous molecular differences influenced by HPV infection. Moreover, patients with HPV-positive and HPV-negative HNSCC show different prognoses, with better outcomes for HPV-positive and worse outcomes for HPV-negative individuals [5].

Characteristic features of HNSCC include a high propensity for metastasis and recurrence, as well as poor therapeutic efficacy due to the frequent resistance of these tumors to standard treatment. Current therapeutic strategies achieve an average five-year survival rate of approximately 50%. Furthermore, about 30% of treated patients exhibit inadequate responses to therapy, which often results in disease recurrence [1].

The American Society of Clinical Oncology has issued diagnostic guidelines for HNSCC [6]. According to these recommendations, diagnosis should include a thorough medical history and ENT examination (including narrow-band imaging-assisted endoscopy), fine-needle aspiration biopsy, histopathological analysis, HPV and EBV testing, and imaging studies such as CT (computed tomography) and PET (positron emission tomography) scans. If the primary tumor remains unidentified, panendoscopy with directed biopsies is recommended. This approach aims to achieve a highly accurate diagnosis and precise localization of the tumor [7].

Despite these efforts, diagnosing HNSCC remains challenging, particularly in the early stages of disease progression, due to the absence of a standardized, effective diagnostic strategy. Additionally, the significant heterogeneity of HNSCC—manifested in anatomical location and etiological diversity—further complicates early detection [6]. Ongoing research is focused on the development of novel diagnostic, predictive, and prognostic biomarkers [7,8]. However, HNSCC involves numerous alterations in molecular signaling pathways, which significantly complicates biomarker development. Most patients are diagnosed at an advanced stage of the disease; only about one-third are diagnosed early and thus have a better prognosis. Unfortunately, reliable biomarkers for early diagnosis and treatment response prediction have yet to be successfully developed and validated, limiting the use of targeted therapies and disease monitoring [5].

The current standard of care for HNSCC is multidisciplinary, involving surgical intervention, systemic drug therapy, and radiotherapy. HNSCC tumors are often resistant to chemotherapy, a phenomenon attributed to the abnormal regulation of cellular pathways that are not yet fully understood, which hinders the development of more effective treatments [9]. One of the major therapeutic challenges is the phenomenon of “field cancerization,” which involves the presence of extensive fields of genetically altered epithelial cells, increasing the risk of disease recurrence due to the incomplete removal of altered tissues [5]. These challenges significantly impair the treatment of HNSCC [9]. Therefore, there is a growing need for more precise therapeutic approaches, with increasing emphasis on combination treatment regimens that include drug therapy, radiotherapy, and surgery [9,10].

The standard chemotherapy regimen for HNSCC typically includes cisplatin (CDDP) combined with 5-FU, a protocol established as the gold standard since 1982 [9,10]. Cisplatin is a metal-based compound with poor water solubility but good solubility in dimethylprimonide and N, N-dimethylformamide. It is stable at room temperature and atmospheric pressure (0 °C and 1013.25 hPa). Intracellularly, cisplatin undergoes hydrolysis and binds to nucleic acids and proteins, leading to DNA damage, the inhibition of cell division, and the induction of apoptosis [11]. It also promotes oxidative stress, modulates calcium signaling, and alters gene expression. However, its use is associated with significant toxicities, including nephrotoxicity, hepatotoxicity, karyotoxicity, ototoxicity, gastrointestinal toxicity, bone marrow suppression, allergic reactions, and reproductive toxicity [11].

Another key chemotherapeutic agent is 5-FU, an antimetabolite introduced in 1957. 5-FU is metabolized into FdUMP (5-Fluorodeoxyuridine Monophosphate), which inhibits thymidylate synthase and disrupts de novo pyrimidine synthesis, thereby impairing DNA synthesis and triggering apoptosis [12,13]. Its use is limited by its toxicity profile—about 30% of patients experience severe, potentially life-threatening side effects, including gastrointestinal toxicity, myelosuppression, cardiotoxicity, and neurotoxicity [14].

In addition to cisplatin and 5-FU, other drugs such as carboplatin and taxanes (e.g., docetaxel and paclitaxel) are used in HNSCC treatment. Cisplatin is also frequently combined with radiotherapy in a regimen known as chemoradiotherapy [9].

Targeted therapy represents a modern medical approach to HNSCC. Due to its precision and selectivity for specific molecular targets, this strategy is increasingly adopted. Targeted therapies may reduce systemic toxicity and are often used in conjunction with standard treatments (chemotherapy or radiotherapy) to improve efficacy. In HNSCC, targets include the epidermal growth factor receptor (EGFR), phosphoinositide-3 kinase (PI3K), human epidermal growth factor receptor 2 (HER2), and vascular endothelial growth factor receptor (VEGFR). Precision medicine allows for the modulation of processes like cell migration, cell cycle regulation, and apoptosis, creating numerous opportunities for enhancing both novel and traditional oncology drugs [15,16].

Immunotherapy is rapidly advancing in oncology, as evidenced by the growing number of clinical and preclinical trials [17]. A key focus is on immune checkpoint inhibitors, especially drugs targeting PD-1 and PD-L1 [17,18]. These therapies work by reactivating T-cell responses against tumor cells, overcoming the immunosuppressive microenvironment characteristic of HNSCC—often described as a “cold tumor” due to a low infiltration of T and NK (natural killer) cells [19]. Currently, checkpoint inhibitors are used as first-line treatment for patients with poor responses to chemotherapy [18]. However, their effectiveness is limited to less than 20% of such cases [17]. Despite this, their mechanism of action holds significant promise for developing new therapies for recurrent HNSCC. Researchers also suggest that progress in immunotherapy may lead to the discovery of novel biomarkers useful for HNSCC diagnosis [19].

## 2. Chemotherapy

**Figure 1:**
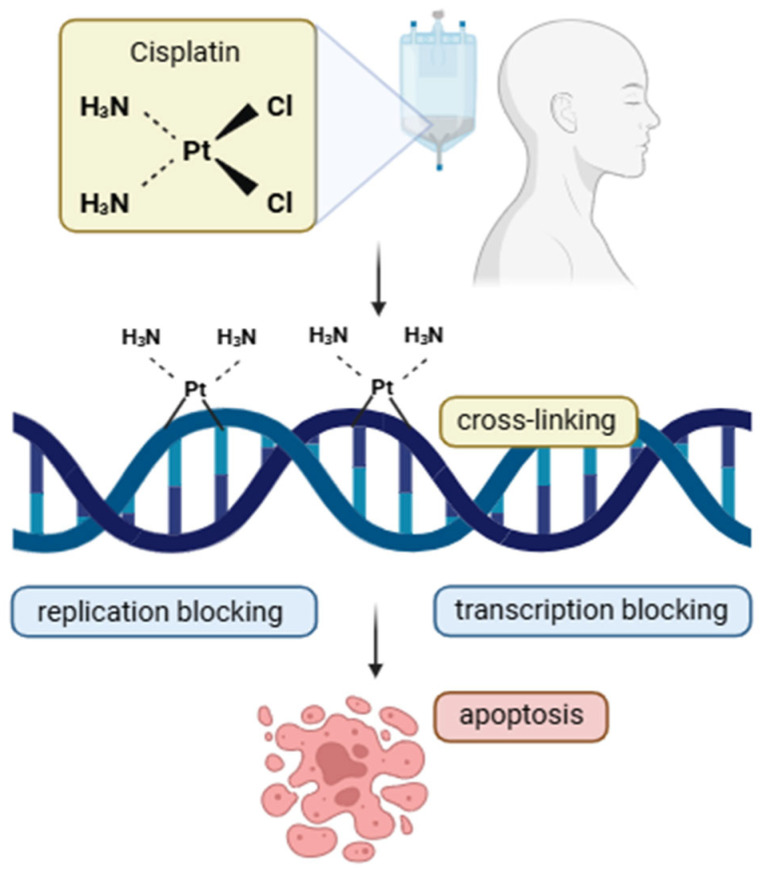
**Mechanism of action of cisplatin.** Cisplatin enters the cell and forms covalent bonds with DNA, primarily at guanine residues, leading to intra- and inter-strand crosslinks. These DNA lesions block replication and transcription processes, ultimately triggering apoptosis.

Chemotherapy remains a frontline treatment for many cancers, despite the development of numerous novel therapeutic strategies [20]. For HNSCC, local tumor resection followed by chemotherapy combined with radiotherapy is commonly employed [21]. Chemotherapeutic agents are classified based on their mechanisms of action (Table 1). Alkylating agents inhibit cancer cell growth by disrupting DNA synthesis through the transfer of alkyl groups to guanine residues. In HNSCC, cisplatin is the most frequently used alkylating agent, with other platinum-based drugs such as carboplatin and oxaliplatin, nitrogen mustards, oxazaphosphorines, and hydrazine also in this category [22]. Since the CDDP in the 1970s, cytotoxic chemotherapy has formed the cornerstone of treatment for HNSCC. Currently, both cisplatin and carboplatin remain key agents in the management of this disease. They are employed in combination with radiotherapy (chemoradiotherapy, CCRT) for patients with locally advanced tumors, as well as in systemic therapy for those with recurrent or metastatic (R/M) HNSCC. Cisplatin continues to be the standard of care for chemoradiotherapy in locally advanced HNSCC, and its general mechanism of action is illustrated in Figure 1.

Numerous clinical trials have demonstrated that its use in combination with radiotherapy significantly improves overall survival compared to radiotherapy alone. However, cisplatin therapy is associated with substantial adverse effects, including nephrotoxicity, neurotoxicity, and ototoxicity, which limit its use in patients with renal impairment or other comorbidities [9,23]. In such cases, carboplatin is a valuable alternative. It is particularly useful for patients in whom cisplatin is contraindicated due to adverse effects or comorbidities. Studies have shown that chemoradiotherapy with carboplatin yields outcomes comparable to cisplatin-based treatment in terms of local disease control, progression-free survival, and overall survival. Notably, the carboplatin-treated group included a higher proportion of elderly patients and individuals with aggressive tumor phenotypes. The most favorable toxicity profile of carboplatin, especially its lower risk of nephrotoxicity and neurotoxicity, makes it a valuable option for patients with additional risk factors [10,24].

In recent years, new therapeutic regimens based on carboplatin have been developed. A retrospective analysis by Carinato et al. [25] evaluated the efficacy and tolerability of a combination therapy consisting of carboplatin, paclitaxel, and cetuximab in patients with recurrent or metastatic HNSCC who were ineligible for cisplatin treatment. The results demonstrated high antitumor activity of this regimen, evidenced by complete and partial responses, along with a favorable safety profile. Such combinations may represent an important treatment option for patients with limited suitability for conventional platinum-based therapies [25,26].

Comparisons between carboplatin and cetuximab have shown that chemoradiotherapy with carboplatin is associated with approximately 15% improved overall survival, underscoring its value as an alternative, especially when cisplatin is not feasible. In a study comparing 131 patients treated with cisplatin to 45 patients who received carboplatin due to contraindications, no significant differences were observed in disease control, progression-free survival, overall survival, or toxicity. Moreover, patients treated with carboplatin, despite being older and having more aggressive tumors, were more likely to complete therapy as planned. Therefore, carboplatin represents a reasonable alternative for patients unable to tolerate cisplatin [27]. The implementation of platinum-based regimens is recommended in both the definitive and adjuvant treatment of locally advanced head and neck cancers. Carboplatin, due to its radiosensitizing properties and lower impact on renal function, is endorsed by the National Comprehensive Cancer Network as an effective alternative for patients unsuitable for cisplatin therapy. Phase II studies have confirmed that carboplatin achieves overall response rates comparable to cisplatin (65–70%) [27]. Carboplatin-based regimens are well-tolerated and demonstrate superior outcomes compared to radiotherapy alone. The combination of carboplatin and paclitaxel has also shown efficacy in other cancers, such as esophageal and lung cancers, where carboplatin acts as a radiosensitizer. In summary, initiating treatment with cisplatin is recommended for patients with good performance status. Carboplatin combined with paclitaxel represents an important alternative for patients who cannot tolerate cisplatin, although it is associated with a risk of hematologic toxicity and a higher likelihood of treatment discontinuation. However, findings from retrospective studies need to be validated in prospective clinical trials [28].

The increasing number of elderly patients with HNSCC poses an additional therapeutic challenge. Although cisplatin combined with radiotherapy remains the standard treatment for locally advanced disease, chemotherapy practices in older patients are variable. In those who tolerate cisplatin, single-dose administration may be considered, as it holds prognostic significance [29].

Recent retrospective and phase IV studies, including KEYNOTE-B10, indicate that combinations of carboplatin with paclitaxel and immunotherapy (e.g., pembrolizumab) demonstrate promising antitumor activity and favorable tolerability, offering new treatment options for recurrent and/or metastatic HNSCC [30].

Clinical summaries indicate that cisplatin is more effective but also more toxic and is preferred in patients with good performance status. Carboplatin is an effective alternative with lower toxicity, used in patients who are unable to tolerate cisplatin. Regimes combining carboplatin with paclitaxel and radiotherapy yield favorable outcomes supported by clinical evidence. Ongoing studies are investigating combination therapies with immunotherapy and targeted treatments, which may enhance efficacy and tolerability. Another treatment strategy for head and neck cancers involves antimetabolites. These compounds disrupt DNA synthesis by inhibiting enzymes essential for replication, including ribonucleotide reductase, dihydrofolate reductase, and thymidylate synthase [21].

**Figure 2:**
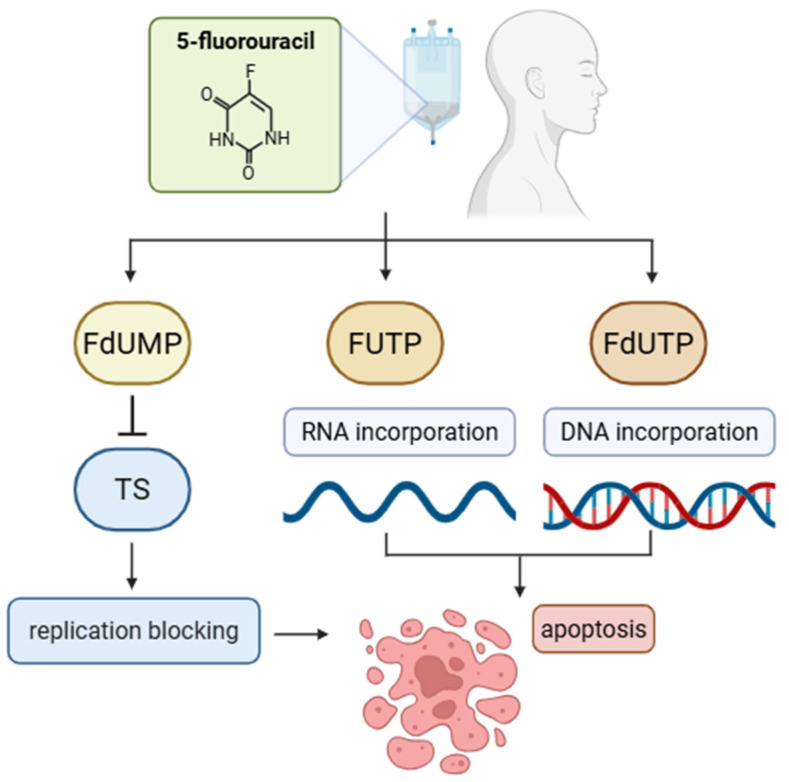
**Mechanism of action of 5-fluorouracil (5-FU).** 5-Fluorouracil is metabolized into several active forms, including fluorodeoxyuridylate (FdUMP), fluorouridine triphosphate (FUTP), and fluorodeoxyuridine triphosphate (FdUTP). FdUMP inhibits thymidylate synthase (TS), leading to the disruption of DNA synthesis and replication blocking. FUTP is incorporated into RNA, impairing its processing and function, while FdUTP is misincorporated into DNA. These combined effects lead to the apoptosis of rapidly dividing cells.

5-FU is the main antimetabolite used in combination therapies for various cancers, including HNSCC [31]. Inside cancer cells, 5-FU is metabolized into FdUMP, FUTP (fluorouridine triphosphate), and FdUTP (fluorodeoxyuridine triphosphate). FdUMP inhibits thymidylate synthase, while FUTP and FdUTP are incorporated into RNA and DNA, respectively, causing nucleic acid damage and leading to apoptosis [32,33]. The general mechanism of action of 5-FU is illustrated in Figure 2. However, 5-FU-based therapies pose challenges, including prolonged infusion times, gastrointestinal and cardiovascular toxicity, and high treatment costs, prompting the development of combination strategies [30].

Topoisomerase I and II inhibitors are also used in chemotherapy, acting by halting the activity of these enzymes critical for DNA replication, leading to strand breaks. Examples include topotecan, idarubicin, daunorubicin, and doxorubicin [22]. Additionally, mitotic spindle inhibitors prevent microtubule formation, causing cell division arrest and eventual cell death. Docetaxel is the most common spindle inhibitor in HNSCC treatment [34]. It targets α/β-tubulin, inhibiting microtubule depolymerization, causing G2/M phase arrest, and inducing apoptosis [35]. Studies on tongue squamous cell carcinoma cell lines showed increased reactive oxygen species (ROS) production, caspase-3 activation, and gasdermin E (GSDME) cleavage by docetaxel (this chemotherapeutic agent promotes apoptosis via the ROS/caspase-3/GSDME pathway) [36]. Additional effects included mitochondrial membrane potential reduction, decreased Bcl-2 expression, increased cytosolic cytochrome c and Bax protein, and the upregulation of Bax, Bak, Bad, and p53 genes—highlighting a multifaceted apoptotic mechanism [35,36].

**Table 1:** Characteristics of key chemotherapeutic drugs used in HNSCC.

Drug Name	Category	Subcategory	Mechanism of Action	Dose of Drug by Body Surface Area (BSA)	Primary Site of Drug Metabolism	References
Cisplatin	Cytotoxic antineoplastic agents	Platinum-based	Cisplatin is a platinum compound that forms covalent DNA crosslinks in cancer cells, blocking replication and transcription. This triggers repair mechanisms that, if overwhelmed, induce apoptosis. It acts through a classical alkylating agent mechanism with notable effectiveness in tumors.	200 mg/m^2^—Cumulative dose of cisplatin (CRT schema) 100 mg/m^2^—Triweekly high dose of cisplatin (CRT schema) 40 mg/m^2^—Weekly dose of cisplatin (CRT schema)	Kidney	[8,37,38]
Carboplatin	Cytotoxic antineoplastic agents	Platinum-based	Carboplatin, a cisplatin analog, forms DNA crosslinks in cancer cells but has lower chemical reactivity. It causes less nephro- and neurotoxicity but greater myelosuppression.	100 mg/m^2^—Weekly dose of carboplatin (CRT schema)	Kidney	[38,39]
5-Fluorouracil	Antimetabolite	Pyrimidine analog	Inhibits thymidylate synthase, blocks DNA synthesis; incorporates into RNA, disrupts RNA function.	250 mg/m^2^—earlier standard dose of 5-FU (per 5 days) 1000 mg/m^2^—standard dose of 5-FU (per 4 days)	Liver	[32,33,40,41]
Docetaxel	Antimitotic agent	Taxane	Stabilizes microtubules, prevents their depolymerization, and arrests cell division in mitosis.	75 mg/m^2^—standard dose of docetaxel in combination chemotherapy (TPF)	Liver	[35,42]

Chemotherapy is a principal approach for treating solid tumors, either as monotherapy or combined with surgery, radiotherapy, immunotherapy, biological therapy, and hormone therapy [42]. Combination therapies are especially effective in HNSCC [43]. Doublet and triplet regimens are commonly used (Table 2) [35]. Established regimens include DC (cisplatin and docetaxel) and PF (cisplatin and 5-FU). Platinum-based combinations are standard for recurrent and/or metastatic HNSCC. In advanced salivary gland cancer, cisplatin is more effective than carboplatin, which shows no phase II activity [34]. Triplet therapies, such as TPF (cisplatin, 5-FU, docetaxel), show superior outcomes to doublet PF regimens [44].

TPEx (cisplatin, docetaxel, cetuximab) is a first-line treatment for recurrent/metastatic HNSCC, as is the EXTREME regimen (cisplatin, docetaxel, 5-FU). TPEx shows similar efficacy to EXTREME with lower toxicity and shorter treatment duration [45].

**Table 2:** Common Chemotherapy Protocols for HNSCC.

Type of Combination Therapy	Drugs	Indication	References
DC	Cisplatin, docetaxel	First-line treatment of recurrent/metastatic HNSCC	[46]
PF	Cisplatin, 5-FU	Induction therapy	[47]
TPF	Cisplatin, 5-FU, docetaxel	Induction therapy	[44]
TPEx	Cisplatin, cetuximab, docetaxel	First-line treatment of recurrent/metastatic HNSCC	[48]
EXTREME	Cisplatin, 5-FU, cetuximab	First-line treatment of recurrent/metastatic HNSCC	[49]

5-FU—5-fluorouracil; HNSCC—head and neck squamous cell carcinoma.

Standard chemotherapy has limitations, including uncontrolled drug release, high systemic toxicity, short half-life, poor solubility, and drug resistance. Therefore, advanced drug delivery systems are promising directions for research.

The primary objective of chemotherapy is to eliminate cancer cells and prevent their further dissemination. While this therapeutic approach can be highly effective, it is also associated with a broad spectrum of adverse effects. These toxicities result from the non-selective mechanism of action of chemotherapeutic agents, which target not only neoplastic cells but also rapidly dividing healthy cells of the host organism [50]. Among the most frequently reported side effects of chemotherapy are nephrotoxicity, often related to agents eliminated renally, cardiotoxicity, hepatotoxicity, neurotoxicity, ototoxicity, hematologic toxicity, cancer-related fatigue (CRF), nausea and vomiting, diarrhea, and alopecia [49,51,52,53]. Despite these challenges, chemotherapy remains a fundamental component of oncologic treatment, and current medical research continues to explore novel strategies to mitigate toxicity and improve patients’ quality of life.

In order to enhance both the efficacy and safety of chemotherapy in the treatment of HNSCC, significant advancements are required in several critical domains. At present, one of the principal limitations of chemotherapy is its systemic toxicity. There is a pressing need for the development of less toxic treatment regimens or more efficient strategies for the prevention and management of adverse effects, such as improved renal protection in patients receiving cisplatin-based therapy [50]. Furthermore, chemotherapy continues to be administered in a relatively non-individualized manner, typically based on patient age, body surface area (BSA), or disease stage [54]. A shift toward treatment personalization—particularly through the use of tumor-specific biomarkers—could enhance therapeutic outcomes and reduce the risk of drug resistance [55]. Drug resistance, whether intrinsic or acquired, remains a major obstacle to the long-term success of chemotherapy. In HNSCC, resistance to commonly used agents such as cisplatin and 5-fluorouracil often leads to treatment failure and limited subsequent therapeutic options. Therefore, further investigation into the molecular mechanisms driving chemoresistance is warranted, with the aim of developing novel treatment strategies capable of overcoming this challenge [56,57,58].

Nanotherapy has shown potential for treating HNSCC [44,59]. Common nanomaterials for anticancer drug delivery include liposomes, micelles, metallic nanoparticles, monoclonal antibody nanoparticles, and polymeric nanoparticles [60,61]. Laser-activated gold nanorods combined with docetaxel were effective in vitro at lower doses [62]. A nanoparticle hydrogel (PTX-CDDP-PH) was used to deliver cisplatin and paclitaxel in mucosal head and neck cancers, prolonging drug release and promoting cell cycle arrest, DNA damage, and apoptosis, with reduced systemic absorption [63]. Combining nanoparticle drug delivery with combination therapies also shows promise. Gold nanoparticles loaded with TPF demonstrated greater cytotoxicity than conventional TPF regimens, suggesting nanotherapy’s promising role in targeted delivery and dose reduction [44].

In summary, chemotherapy remains a cornerstone in the treatment of HNSCC. Due to its high efficacy, cisplatin continues to be regarded as the gold standard; however, its considerable toxicity limits its use in patients with comorbidities. Carboplatin represents a viable alternative, offering a more favorable safety profile, albeit with slightly reduced cytotoxic potency. Despite therapeutic advances, chemotherapy is still associated with numerous adverse effects, including nephrotoxicity, neurotoxicity, and ototoxicity, which compromise treatment tolerability in many patients. Both intrinsic and acquired resistance to chemotherapy remain among the most significant challenges, often restricting further therapeutic options. As a result, modern chemotherapy increasingly relies on the complex molecular mechanisms of cytotoxic agents, whose efficacy may be enhanced through combination strategies and the use of nanocarrier systems that enable targeted drug delivery to cancer cells.

## 3. Immunotherapy

Immunotherapy is an innovative form of treatment that aims to activate the body’s natural defense mechanisms in the fight against cancer. Unlike chemotherapy, it does not directly attack cancer cells but supports the immune system in identifying and eliminating them. A key role in this process is played by T lymphocytes and antigen-presenting cells (APCs), which initiate and regulate the immune response within the tumor. Immunotherapy focuses on developing drugs that can modulate the activity of these cells in the tumor microenvironment [64].

One of the breakthroughs in the development of immunotherapy was the approval in 2011 by the US Food and Drug Administration (FDA) of ipilimumab—a monoclonal antibody directed against the cytotoxic T-lymphocyte-associated protein 4 (CTLA-4) receptor—which became the first approved immunotherapeutic drug for the treatment of metastatic melanoma [65]. Even earlier, in 1992, Honjo’s team first identified the PD-1 gene, and shortly afterwards, its ligand, PD-L1, was discovered. These discoveries gave rise to a new generation of immuno-oncology drugs that, by blocking immune checkpoints, enable T lymphocytes to fight cancer cells more effectively [66]. Currently, PD-1 and PD-L1 inhibitors are gaining increasing importance as effective therapeutic options in the treatment of many different cancers. They are used, among others, in the treatment of metastatic melanoma, non-small-cell and small-cell lung cancer, triple-negative breast cancer, platinum-resistant pancreatic and ovarian cancers, as well as in cervical cancer, renal cell carcinoma, gastric and gastroesophageal junction adenocarcinoma, colorectal cancer, hepatocellular carcinoma, and prostate cancer [65].

**Figure 3:**
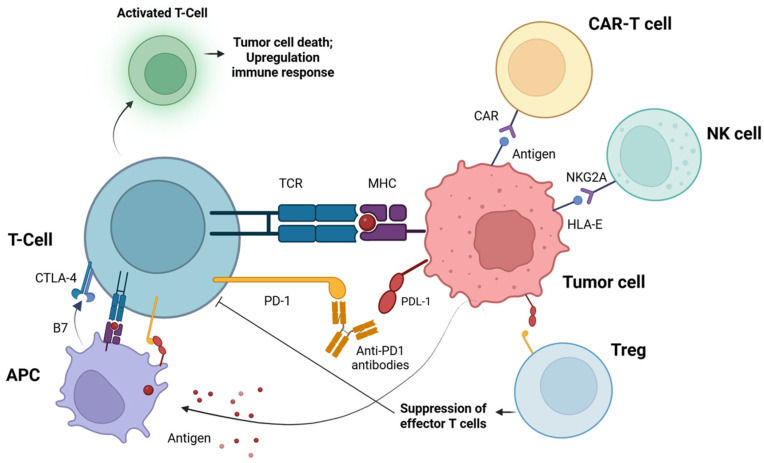
In the immune response, tumor antigens are engulfed by antigen-presenting cells (APCs), which then present these antigens to T lymphocytes via major histocompatibility complex (MHC) molecules. This leads to T-cell activation and increased expression of the programmed cell death protein 1 (PD-1) receptor on their surface. Tumor cells, through the expression of PD-L1, bind to PD-1 on the surface of activated T cells, resulting in the inhibition of their function and the suppression of the antitumor immune response. PD-L1 can also engage PD-1 on regulatory T cells (Tregs), further contributing to immunosuppression by raising the activation threshold of effector T cells. Antibodies such as pembrolizumab (PD-1 inhibitors) have a high affinity for PD-1 and can compete with endogenous PD-L1. By blocking the interaction between PD-1 and its ligands, they restore T-cell activity and enhance the body’s immune response to cancer cells. In addition to the PD-1/PD-L1 blockade, immunotherapy strategies in head and neck squamous cell carcinoma (HNSCC) have also targeted cytotoxic T-lymphocyte-associated antigen 4 (CTLA-4) on T cells, natural killer (NK) cell receptors such as NKG2, and adoptive cell therapies including chimeric antigen receptor (CAR) T-cell therapy.

Immunotherapy has revolutionized the approach to the treatment of HNSCC, especially in recurrent or metastatic cases. In the spotlight are antibodies blocking immune checkpoints (so-called checkpoint inhibitors, ICIs). These drugs work by modifying T cell responses—controlling their activation to prevent excessive inflammation while enhancing the effectiveness of the anticancer immune response [65]. The mechanism of action of the PD-1 inhibitor and its role in the body’s immune response against cancer cells are shown schematically in Figure 3.

Continuing the development of immunotherapy for HNSCC, a landmark moment came with the FDA approval in 2016 of two immune checkpoint inhibitors: pembrolizumab and nivolumab. Both drugs, which are antibodies directed against the PD-1 receptor, are approved for use in patients with recurrent or metastatic HNSCC whose disease has progressed during or after treatment with platinum-based chemotherapy [67]. Pembrolizumab and nivolumab improve survival compared with investigator’s choice chemotherapy [68,69].

Mentioned earlier, pembrolizumab has played a key role in the treatment of recurrent and metastatic HNSCC, and its efficacy has been extensively studied in the KEYNOTE clinical trial program. Three pivotal trials—KEYNOTE-012 (Phase I, completed in 2016), KEYNOTE-055 (Phase II, completed in 2017), and the landmark Phase III trial KEYNOTE-048 (completed in 2019)—provided solid evidence for the use of pembrolizumab in this patient population. Results showed a significant improvement in overall survival (OS) and a favorable safety profile compared with conventional chemotherapy [64].

In addition, pembrolizumab has also shown promise in the perioperative setting. Uppaluri et al. [70] reported that a single dose of pembrolizumab in patients with high-risk disease led to a pathological tumor response of >50% in 22% of patients, and the annualized relapse rate was only 16.7%, which is a significant improvement over historical data (~35%) [70].

The largest study of neoadjuvant and adjuvant pembrolizumab to date included 92 patients with locally advanced, HPV-negative HNSCC. A significant improvement in 1-year disease-free survival (DFS) was noted in the group of patients with a pathological response (93% vs. 72%), with a hazard ratio of 0.29 for recurrence. In response to these results, the KEYNOTE-689 study (NCT03765918) was initiated to evaluate the efficacy and safety of perioperative pembrolizumab in combination with standard radiotherapy with or without cisplatin in previously untreated patients with respectable HNSCC [71].

The Phase III KEYNOTE-048 trial compared three treatment strategies: pembrolizumab monotherapy, pembrolizumab plus chemotherapy (platinum compounds + 5-FU), and standard chemotherapy plus cetuximab. In the overall group, pembrolizumab plus chemotherapy extended the median OS (overall survival) to 13 months, compared with 10.7 months in the chemotherapy plus cetuximab group (HR 0.77; *p* = 0.0034). In patients with high PD-L1 expression, the benefit was even more pronounced, with pembrolizumab monotherapy providing a median OS of 14.9 months, compared with 12.3 months in the cetuximab group. Even in all PD-L1-positive tumors, pembrolizumab was superior (12.3 vs. 10.3 months) [72].

The Phase IV KEYNOTE-B10 trial also provided important data evaluating the efficacy of a carboplatin- and paclitaxel-based regimen in combination with pembrolizumab in patients with recurrent or metastatic HNSCC. The results were comparable to those from the KEYNOTE-048 trial, suggesting that this regimen may represent a reasonable alternative to the combination of platinum, 5-FU, and pembrolizumab, especially in patients with contraindications to more intensive chemotherapy [73].

The choice between pembrolizumab monotherapy and a combination of treatment with chemotherapy should be individualized—it depends, among others, on the stage of the disease, PD-L1 expression, and patient preferences. The addition of chemotherapy usually increases the objective response rate but may be associated with a higher risk of adverse events. Therefore, the therapeutic decision should be balanced and based on a full clinical evaluation [74].

Despite advances in the treatment of HNSCC with immune checkpoint inhibitors (ICIs), up to 60% of patients do not respond to therapy, and the rate of complete response rarely exceeds 20% [75]. One of the key mechanisms underlying this resistance may be the activity of regulatory T cells (Tregs), which exert immunosuppressive effects within the tumor microenvironment (TME), thereby limiting the efficacy of antitumor immune responses [76].

Tregs, identified by the expression of CD4, CD25, and Foxp3, are essential for maintaining immune tolerance; however, in the context of cancer, their function facilitates immune evasion by tumor cells [77]. In HNSCC, increased infiltration of Tregs within the TME is observed and is associated with poor prognosis and reduced treatment efficacy. Treg-mediated immunosuppression involves several mechanisms, including the sequestration of IL-2, which limits the proliferation of effector T cells [78]; the secretion of immunosuppressive cytokines such as IL-10, TGF-β, and IL-35 [79]; and the expression of inhibitory molecules, including CTLA-4 and PD-L1 [80]. Additionally, Tregs can induce metabolic dysregulation and exert cytotoxic effects on effector immune cells. Their recruitment to the TME is facilitated by chemokines such as CCL20 and chemokine receptors, including CCR10 and CXCR4 [81]. Within the tumor environment, Tregs undergo phenotypic and functional reprogramming influenced by signaling pathways such as TCR/CD28, PI3K-AKT-mTOR, and HIF-1α, as well as by neuropilin-1 [82]. Tregs suppress not only CD4^+^ and CD8^+^ T cell activity but also that of natural killer (NK) cells and dendritic cells [83]. Their presence further promotes the recruitment of myeloid-derived suppressor cells (MDSCs) and tumor-associated macrophages (TAMs), enhancing the immunosuppressive milieu [84].

The high expression of immune checkpoint molecules such as CTLA-4, PD-1, TIM-3, and PD-L1 on Tregs contributes to the inhibition of antitumor immunity and may limit the effectiveness of immunotherapy [85]. Therefore, effective therapeutic strategies should include the selective depletion or functional reprogramming of Tregs to enhance responses to immunotherapy.

## 4. CDK Inhibitors

A new therapeutic approach in the targeted therapy of HNSCC is the use of cyclin-dependent kinase inhibitors (CDKIs). CDKs are a group of serine/threonine kinases—multifunctional enzymes composed of catalytic and regulatory subunits—that phosphorylate other proteins. They play a key regulatory role in the cell cycle [86]. Their function is strictly dependent on the presence of specific cyclin proteins. CDK1, CDK2, CDK4, and CDK6 are well-described regulators of cell cycle machinery, while other CDKs, including CDK7, CDK9, and CDK12, are primarily involved in transcriptional regulation [86].

CDK4 and CDK6, in particular, serve as key checkpoints for the transition from the G1 to the S phase of the cell cycle. The critical involvement of CDKs in cell proliferation has provided a strong rationale for investigating CDK inhibition as an anticancer strategy [86]. However, depending on the tumor type, specific mutations, and differentiation status, cancer cells do not always respond uniformly or predictably to CDK inhibition [87]. Commonly altered genes include cyclin-dependent kinase inhibitor 2A (CDKN2A) and cyclin D1 (CCND1), both of which are involved in cell cycle regulation. These genes encode a complex set of proteins, including CDKs and cyclin D, that govern progression through the cell cycle. The sustained activation of CDK4/6 is thought to occur in most cancers as a mechanism to promote continuous transition from the G1 to the S phase. In HNSCC, alterations in CDKN2A or CCND1 are two key genomic events that lead to a loss of cell cycle checkpoint control. In patients with HNSCC, CDKN2A alterations include homozygous deletions in approximately 30%, additional gene mutations in 10–20%, and epigenetic silencing in up to 80% of cases. CCND1 is amplified as part of the 11q13 amplicon in about 20% of tumors. Between these two genes, genomic alterations in CDKN2A and CCND1 have been identified in 60–94% of HNSCC cases, making them potential therapeutic targets [88].

However, CDKN2A and CCND1 aberrations do not always translate into increased protein activity of cyclin D1 and CDK4/6. The status of retinoblastoma protein (Rb) phosphorylation may serve as an additional biomarker, alongside CDKN2A/CCND1 genomic aberrations, in future clinical trials of CDK4/6 inhibitors. Moreover, mutations in CDK6 (5%) and RB1 (2%) have also been reported [89].

First-generation cyclin-dependent kinase inhibitors—such as flavopyridol and roscovitine—did not selectively inhibit specific CDKs and produced disappointing outcomes in clinical trials for solid tumors [87]. Initially, their therapeutic effects were believed to result from the inhibition of CDKs involved in cell cycle regulation. However, subsequent studies demonstrated that much of their cellular activity was more likely attributable to the inhibition of CDK7 and CDK9—kinases responsible for regulating RNA transcription during the mitotic phase of the cell cycle—as well as the expression of genes involved in apoptosis [87].

Flavopyridol is the most extensively studied first-generation CDKI. Between 1998 and 2014, more than 60 clinical trials were conducted to evaluate its efficacy. It has been shown to inhibit multiple CDKs, including CDK1, CDK2, CDK4, CDK6, CDK7, and CDK9. Despite its broad and potent activity in vitro, flavopyridol demonstrated only modest efficacy in in vivo studies [87].

The main drawback of the first-generation CDKIs, flavopyridol and roscovitine, was their lack of selectivity with associated side effects in patients, while not being sufficiently effective in preventing tumor progression. Therefore, further development has focused on improving the selectivity of CDKIs [87]. Similarly, clinical trials of second-generation agents (dinaciclib, AT7519, SNS-032, AG-024322) in advanced solid tumors, which selectively acted on CDK1 and CDK2, were similarly poor [86]. Dinaciclib very effectively targeted CDK2 and CDK5 (IC 50 values of 1 nM each compared to 12 and 14 nM for flavopyridol) [87]. In vitro, dinaciclib completely inhibited Rb phosphorylation, which correlated with the onset of apoptosis and the complete inhibition of DNA synthesis in >100 cancer cell lines of different origins and backgrounds. In addition, dinaciclib demonstrated enhanced efficacy in treating solid tumors in a range of mouse models at doses below the maximum tolerated level. The overexpression of CDK2 in neuroblastoma tissue is associated with poor overall survival, suggesting a potential patient selection strategy during the clinical development of this drug [87].

It is now known that cells begin to proliferate earlier in the cell cycle, more specifically in the late G1 phase, which is regulated by CDK4/6. This partly explains the poor results observed with the first CDKIs and soon initiated the development of third-generation CDK4/6 inhibitors (palbociclib, abemaciclib, ribociclib) [86].

Since 2015, four CDK4/6 inhibitors have received approval from the US FDA; namely, palbociclib (PAL), ribociclib, and abemaciclib for the treatment of hormone receptor-positive (HR +), HER2-positive breast cancer, and most recently, trilaciclib as myeloablative therapy for extensive-stage small-cell lung cancer [86].

CDKI was first investigated in a phase I/II study (NCT02101034) of PAL in combination with cetuximab in patients with recurrent/metastatic HNSCC. The results of the phase I study (n = 9) showed that the combination was safe and had antitumor activity. A phase II study showed objective responses in 11/28 (39%, 22–59%) patients with platinum-resistant HNSCC and in 5/27 (19%, 6–38%) patients with cetuximab-resistant HNSCC [86,87,88]. These association effects have been shown to be due to chemotherapy-induced tumor S-phase recruitment/synchronization, resulting in a greater degree of cell cycle arrest and apoptosis. A preclinical study evaluating ribociclib and cisplatin in ovarian cancer models showed significant synergy when administered concomitantly with stem cell reduction in both Rb mutant and wild-type cell lines. Preclinical work on HNSCC cell lines similarly demonstrated the suggested synergistic potential of palbociclib and platinum therapy [88].

**Figure 4:**
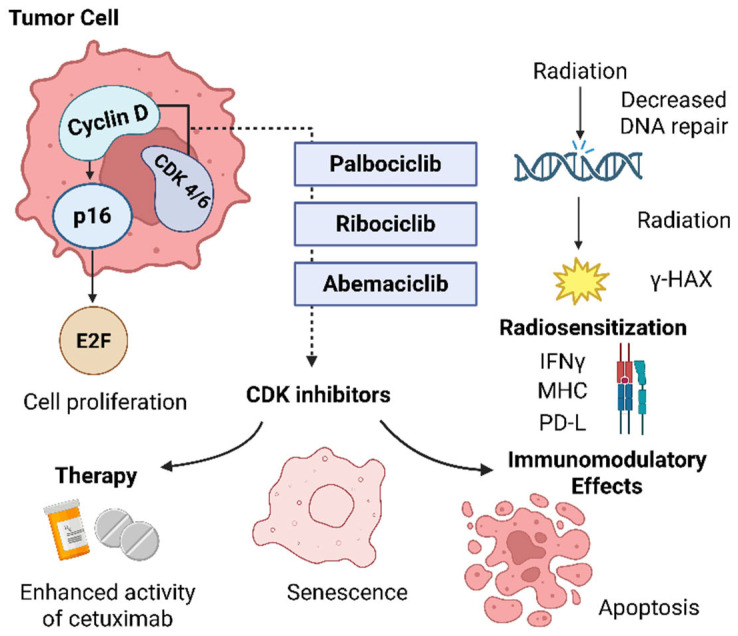
**Mechanism of action of selective CDK4/6 inhibitors in HNSCC:** inhibition of the cyclin D1–CDK4/6 complex prevents cell cycle progression by reducing E2F activity. CDKIs also promote radiosensitization, immune activation, senescence, and apoptosis, and may enhance cetuximab efficacy. **CDK4/6**—Cyclin-dependent kinases 4 and 6, key enzymes that regulate the G1-to-S phase transition of the cell cycle; **cyclin D**—a regulatory protein that activates CDK4/6, frequently overexpressed in HNSC. E2F–E2 promoter-binding factor—A family of transcription factors that drive expression of genes required for S-phase entry and DNA replication; γ-H2AX—gamma-H2A histone family member X—a phosphorylated variant of the H2AX histone; serves as a marker of DNA double-strand breaks and activates DNA damage response pathways; IFN—interferon (typically IFN-γ, interferon-gamma)—a cytokine that activates immune responses, enhances antigen presentation, and promotes MHC expression; MHC—major histocompatibility complex—a group of proteins (Class I and II) that present antigenic peptides on the cell surface for recognition by T cells; p16—cyclin-dependent kinase inhibitor 2A (CDKN2A)—a tumor suppressor protein that inhibits CDK4/6 activity, preventing phosphorylation of Rb and halting the G1-to-S phase cell cycle transition; PD-L1—programmed death-ligand 1—an immune checkpoint molecule that binds PD-1 on T cells, leading to the inhibition of T cell activation and immune evasion by tumor cells.

Palbociclib is the first approved selective CDK4/6 inhibitor that can reverse cetuximab resistance by counteracting the actions of deregulated cyclin D1 (Figure 4) [88,90]. PAL exerts potent antiproliferative effects on Rb-positive cell lines and human breast and colorectal xenografts. PAL decreases Rb phosphorylation and Ki-67 (nuclear antigen) expression in Rb^+^ models but has no activity in Rb^−^ tumor xenografts. Preclinical studies evaluating CDK4/6 inhibitors suggest synergy with chemotherapy for several cancer types [88].

The first clinical trial to evaluate a selective CDK4/6 inhibitor for the treatment of HNSCC was the novel combination of palbociclib and cetuximab, which proved to be fully feasible and safe [91]. The recommended dose of palbociclib is 125 mg daily for 1–21 days at 28-day intervals with cetuximab. A measurable reduction in target lesions was observed in 56% of patients. In patients with platinum-resistant or cetuximab-resistant HNSCC unrelated to HPV, PAL and cetuximab have shown promising activity results [90]. Non-HPV-related HNSCC is caused by the overactivation of the CDK4/6 and cyclin D1 regulatory complex, resulting in cell cycle progression and tumor growth, suggesting that CDK4/6 inhibition may be a rational therapeutic strategy in this case [90]. Further studies of CDK4/6 inhibitors are warranted in non-HPV-related head and neck squamous cell carcinoma [90].

PALATINUS (NCT02499120), a randomized placebo-controlled phase II study of palbociclib in combination with cetuximab for platinum-resistant, non-HPV-related head and neck cancer (n = 125), showed no significant difference in median OS and progression-free survival (PFS) between the palbociclib and placebo groups [86]. Another phase II study (NCT03194373) of palbociclib in combination with carboplatin in patients with inoperable recurrent or metastatic squamous cell carcinoma of the head and neck (n = 21) reported one (6%) partial response, with a median PFS of 2.9 (1.2–13.3) months and significant treatment-related toxicity. Following the approval of pembrolizumab and nivolumab for the treatment of platinum-resistant HNSCC, a number of clinical trials have been conducted on the association of checkpoint immunotherapy with protein kinase inhibitors (NCT03938337, NCT03655444, NCT03498378, NCT04213404) [86].

To this end, there are two challenges for both researchers and clinicians: therapy-related toxicity, given that these agents are often prescribed for long periods of time, and inevitable therapy resistance. Regarding toxicity, it is generally manageable, with hematopoietic side effects being common during treatment with palbociclib and ribociclib, in particular neutropenia (but not with neutropenic fever) and leukopenia, while gastrointestinal side effects and fatigue are more common with abemaciclib [86].

CDKIs did not necessarily work as well as monotherapy. Instead, CDKIs have been shown to be particularly effective in combination with chemotherapy, radio-, or immunotherapy. Such promising combination therapy strategies are of great interest for HNSCC. The specific CDK4/6 inhibitors albociclib, ribociclib, and abemaciclib are the most studied. Unfortunately, approximately 10% of patients will have primary resistance to CDK4/6 inhibitors [87].

## 5. The Role of PARP1 and Its Inhibitor Olaparib in HNSCC

**Figure 5:**
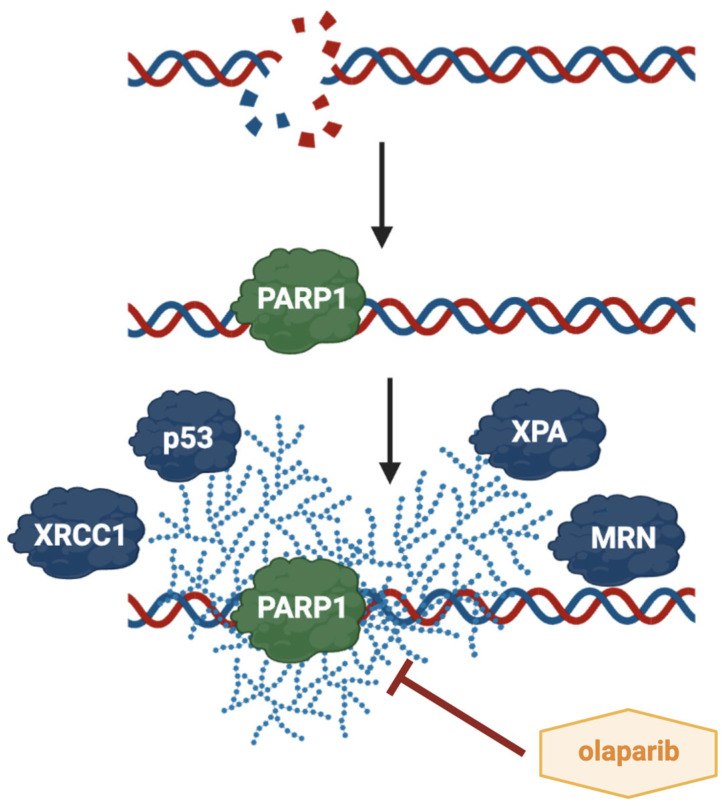
Schematic representation of PARP1 activity in cell nucleus. PARP1 is required for the robust detection of DNA double-strand breaks and, through PARylation, recruits proteins involved in DNA damage response, including p53, XRCC1, and MRN. Olaparib, by inhibiting PARP1 activity, increases DNA damage in HNSCC and may lead to cell death.

Another interesting molecular target is PARP1, a protein that belongs to the PARP superfamily. It catalyzes the polymerization of ADP-ribose units derived from NAD^+^, a process known as PARylation [92]. This post-translational modification involves the covalent attachment of poly (ADP-ribose) (PAR) chains, which significantly alters protein functions [93]. In addition, a variety of proteins, termed PAR-readers, are able to bind to PAR in a non-covalent manner, influencing their localization, stability, and interactions with other macromolecules. When PARP1 detects DNA damage, it induces a conformational change in its autoinhibitory helical domain, facilitating NAD^+^ binding and subsequent conversion to PAR [94]. PARP1 plays a critical role in the detection of double-strand breaks and in initiating the DNA damage response through interactions with MRE11 and ATM (ataxia telangiectasia mutated) [95]. During homologous recombination, PARP1 further recruits MRE11 to the DSB region [96]. PARP1 is also integral to the cellular response to replication stress. The binding of PARP1 to DNA breaks or G-quadruplexes ahead of the replication fork, followed by its auto-PARylation, is essential for stabilizing stalled replication forks and facilitating replication restart [97]. Conversely, insufficient de-PARylation can result in the formation of PARP1 complexes that become barriers to replication [98]. PARP1 directly interacts with key regulatory proteins such as p53 and p21 in response to DNA damage and replication stress, thereby inhibiting DNA replication. Specifically, p53 serves as a substrate for covalent PARylation at its C-terminal domain and also engages in a high-affinity, non-covalent interaction with PAR [99]. This non-covalent modification reduces the ability of p53 to bind DNA in a sequence-independent manner, while enhancing its sequence-specific transcriptional activity. Moreover, PARylation disrupts the interaction between p53 and the nuclear export receptor CRM1, leading to the accumulation of p53 in the nucleus and promoting the upregulation of p21 [100]. Figure 5 presents a simplified illustration of PARP1 activity within the cell nucleus.

Numerous studies have demonstrated that PARP1 expression is significantly upregulated in various cancer types, including breast cancer [101,102], colorectal cancer [103], prostate cancer [104,105], and glioma [106]. Notably, elevated PARP1 expression has also been observed in HNSCC [107]. In HNSCC, the number of PARP1-positive tumor cells varies depending on tumor localization, with the highest levels found at the invasive margins rather than in the tumor core. Interestingly, precancerous lesions (e.g., severe dysplasia or carcinoma in situ) also exhibit high PARP1 expression levels [106]. It is believed that PARP1 overexpression in HNSCC results primarily from increased DNA damage in genetically unstable cancer cells, rather than from the activation of specific oncogenic pathways [101]. Importantly, elevated PARP1 levels in primary oral cancer cells have been linked to therapy resistance. Recurrent cancer cells are highly dependent on PARP1 activity for survival under treatment pressure. Furthermore, in these recurrent cancer cells, cisplatin and 5-fluorouracil have been shown to induce PARP1 gene expression [107].

In this context, PARP1 inhibition appears to be a promising therapeutic strategy in cancer treatment, particularly in HNSCC. Among commercially available PARP inhibitors, olaparib is one of the most widely used. It is rationally designed to act as a competitive NAD^+^ inhibitor at the catalytic site of both PARP1 and PARP2. Olaparib selectively induces cell death in homologous recombination (HR)-deficient cancer cells, leveraging the concept of “synthetic lethality” [108,109]. HR-deficient cells are thought to be sensitive to PARP inhibition due to the accumulation of single-strand DNA breaks, which, in the absence of PAR synthesis, lead to replication fork collapse and double-strand breaks that require HR machinery for repair [110]. The therapeutic effect of PARP1 inhibition has also been observed in the context of radiotherapy. Radiosensitization by olaparib has been reported in preclinical models across several cancer types [111,112], although it is largely dependent on the proportion of cells in the S phase of the cell cycle [113]. However, HR gene mutations are rare in many tumors, and not all mutation carriers respond to PARP inhibitor monotherapy [114]. As such, an increasing number of studies propose combining olaparib with other therapeutic agents. For instance, the combination of olaparib and cetuximab (an EGFR inhibitor) with irradiation in HNSCC cells results in greater DNA damage than either agent alone. The EGFR signaling pathway is involved in the repair of DNA double-strand breaks through DNA-PK activation in the nucleus [115]. Cetuximab disrupts EGFR activity and radiation-induced nuclear import [116]. The triple combination of cetuximab, olaparib, and irradiation leads to enhanced apoptosis, increased p21 expression, and greater clonogenic cell death in HNSCC cells. Moreover, the simultaneous targeting of DNA-PK (a downstream target of EGFR) and PARP1 in lung cancer cell lines has been shown to induce senescence [116]. The potential of olaparib in combination therapies has also been evaluated in chemotherapy settings, although PARP1 inhibition combined with histone lysine demethylase (KDM) inhibitors alone did not yield substantial efficacy in HNSCC cells. However, the addition of cisplatin significantly enhanced therapeutic outcomes. Dual combinations of KDM inhibitors (particularly JIB-04 and GSK-J4) with cisplatin, and triple combinations involving cisplatin and olaparib, exhibited increased cytotoxic and pro-apoptotic activity, suggesting a promising therapeutic approach for HNSCC [117]. These results highlight the chemosensitizing potential of KDM inhibitors and their synergy with olaparib [118].

Another important risk factor in oropharyngeal squamous cell carcinoma is infection with HPV [119]. PARP1 is upregulated in HPV-positive tumors, driven by the oncogenic proteins of HPV16. HPV-positive HNSCC cell lines show higher sensitivity to both olaparib and cisplatin compared to HPV-negative lines. Furthermore, the combination of cisplatin and olaparib synergistically reduces cell viability across all tested HNSCC lines, regardless of HPV status [119], suggesting a broadly effective therapeutic combination. This synergy enables drug dose reduction, potentially decreasing side effects while enhancing therapeutic efficacy [120].

Noteworthily, non-invasive imaging of PARP1/2 using PET is also under development. Several radiolabeled PARP inhibitors have been evaluated in preclinical studies [121], demonstrating a correlation between tracer uptake and PARP1 expression [122]. Importantly, low non-specific uptake in healthy head and neck tissue has been reported. Schöder et al. [123] performed clinical imaging using ^18^F-PARPi, a PARP1/2 inhibitor based on the olaparib scaffold [124], in HNSCC patients. The administration of ^18^F-PARPi was well-tolerated and shows promise as a novel diagnostic agent for imaging head and neck squamous cell carcinoma [123,125].

To summarize, the inhibition of PARP1 activity—particularly via olaparib—emerges as a compelling addition to existing therapeutic regimens for HNSCC. Due to its versatility, olaparib can be effectively combined with diverse treatment modalities, significantly enhancing its efficacy. This strategy holds considerable potential to improve patient prognosis in the future.

## 6. Potential Role of New Molecular Targets

Biomarkers can confirm the presence of a specific disease entity (diagnostic), predict disease progression (prognostic), or predict the effect of a given treatment (predictive). Currently, the most frequently cited diagnostic and prognostic biomarkers for HNSCC are HPV DNA/mRNA, HPV-E6 serotype, HPV ctDNA, EBV RNA, and p16^Ink4a. Prognostic biomarkers include estrogen receptor positivity, hypoxia markers, TP53, P53, CCND1, Cathepsin-D, BCL2, and VEGF. Biomarkers that are both prognostic and predictive include APOBEC (Apolipoprotein B mRNA Editing Enzyme, Catalytic Polypeptide), the neutrophil-to-lymphocyte ratio, PTEN, ERCC1, PD-L1, and EGFR. In addition to the aforementioned biomarkers, a large number of potential candidates have been described in the literature. However, the development of useful biomarkers often encounters challenges such as low sensitivity, inconsistency in study results, and the need for further research to confirm their effectiveness in targeted therapies [8,126,127,128,129,130].

Despite advances in oncological therapies, significant limitations remain in the diagnosis and monitoring of treatment outcomes, particularly in HNSCC. The lack of specific diagnostic markers hampers early detection, emphasizing the need for more selective biomarkers to improve treatment strategies [7,8,126,127]. Two promising candidates have recently emerged: nicotinamide N-methyltransferase (NNMT) and paraoxonase 2 (PON2).

NNMT is a cytosolic enzyme that catalyzes the methylation of nicotinamide (vitamin B3) to 1-methylnicotinamide (MNA), using S-adenosyl-L-methionine (SAM) as a methyl donor [130]. Its activity alters the SAM:SAH ratio, leading to global hypomethylation and potential epigenetic dysregulation, which has been implicated in tumor progression [131]. NNMT is mainly expressed in the liver and white adipose tissue but is also upregulated in tumor cells and cancer-associated fibroblasts (CAFs) [132]. Differential NNMT expression between a tumor and its surrounding tissue has been proposed as a prognostic marker in several cancers, including oral squamous cell carcinoma, lung adenocarcinoma, and hepatocellular carcinoma [132,133], and is also elevated in HNSCC.

Therapeutic interest in NNMT has focused on its inhibition, particularly using bisubstrate inhibitors that mimic NA and SAM. These compounds demonstrated high biochemical potency (IC_50_~3.7 nM) but limited cytotoxicity in cell lines due to poor membrane permeability [134]. Similar limitations were observed in prodrug forms requiring high intracellular concentrations to achieve functional effects [131]. Nonetheless, NNMT inhibitors effectively reduced MNA levels in vitro [131,134]. These findings suggest that delivery strategies, such as nanocarriers, may be necessary to enhance therapeutic efficacy. Moreover, NNMT inhibition has shown potential in sensitizing cancer cells to chemotherapy and radiotherapy [135].

PON2, an intracellular membrane-associated enzyme from the paraoxonase family, is ubiquitously expressed across human tissues [136]. The overexpression of PON2 has been reported in various cancers, where it contributes to cell survival, chemoresistance, and anti-apoptotic mechanisms [137,138]. Silencing PON2 sensitizes cancer cells to chemotherapy and inhibits invasion and migration.

In a study of squamous cell carcinoma (including HNSCC), elevated PON2 expression was observed in tumor tissues, correlating with malignancy grade but not with patient age, sex, or tumor differentiation [136]. Additionally, PON2 knockdown enhanced cisplatin sensitivity in oral cancer cell lines, while overexpression increased resistance. Oxidative damage upon PON2 silencing was confirmed via FTIR microscopy, supporting its role in chemoresistance [137]. These findings position PON2 as a potential diagnostic, prognostic, and therapeutic target in HNSCC.

## 7. Clinical Trials and Direction of Molecular Therapies

We searched the ClinicalTrials.gov database using the following filters: Condition/Disease —“HNSCC”, Sex—“All”, and a study start date range from 1 January 2020 to 31 December 2025. A total of 825 results were obtained, covering clinical trials in various phases. This high number of registered studies in recent years indicates intensive efforts to develop new treatment methods for HNSCC. After narrowing the search criteria to selected molecular pathways— phosphoinositide 3-kinase (PI3K), EGFR, PD-1, CDK4, and PARP—while maintaining the other parameters, the following numbers of active clinical trials were identified: 6, 58, 186, 7, and 4, respectively. Based on these data, it can be concluded that the greatest research interest in recent years has focused on immunotherapy targeting the PD-1 pathway. Other recent clinical trials in the field of targeted therapies of HNSCC are presented in Table 3 to show the present direction of research.

PI3K belongs to a family of enzymes that play a major role in cell growth, proliferation, and differentiation, which translates into tumor development and progression. PI3K interacts with protein kinase B (Akt) and mammalian target of rapamycin (mTOR) to form the PI3K/Akt/mTOR axis. This pathway is overactive in 90% of HNSCC cases, and its increased activity results in a reduced tumor response to radiotherapy, cytostatics, and hormonal therapy [138,139].

Kurupi R. et al. [140] conducted studies on SCC-9, JHU-022, HSC-4, BHY, and BICR22 cell lines treated with an SHP2 inhibitor. They observed that the inhibitor effectively blocked the PI3K and MEK pathways in a large subgroup of HNSCC. The researchers demonstrated that the inhibition of these pathways was mediated by the GAB1 protein, which is directly affected by SHP2. They showed that GAB1 is critical for the survival of HNSCC cells [140].

Dunn L.A. et al. [141] conducted a clinical trial on 11 patients with HNSCC using a combination therapy of a PI3K inhibitor and radiotherapy. One patient who received the level 2 dose of the inhibitor experienced nausea leading to treatment discontinuation. They also found that administration of the level 3 dose led to a decrease in lymphocyte count, stomatitis, dysphagia, hyperglycemia, maculopapular rash, and palmar–plantar erythrodysesthesia syndrome. Ten out of ten evaluable patients remained disease-free after treatment. The researchers concluded that the recommended dose for the PI3K inhibitor is 250 mg/day in combination with cetuximab and radiotherapy [141].

Moreover, preclinical studies indicate that the simultaneous inhibition of EGFR and PI3K in HNSCC cell lines and xenografts leads to the synergistic inhibition of cell proliferation [142].

De Wit J.G. et al. [142] conducted a study on fluorescent molecular imaging using EGFR. In their study, they used the fluorescent marker cetuximab-800CW. They observed that ex vivo imaging with this marker resulted in excellent tumor detection with 100% sensitivity, as well as the identification of most nearby margins. They concluded that the use of this marker enables the accurate determination of tumor margins in oral cancer, which can facilitate surgical correction and reduce the need for adjuvant chemoradiotherapy [142].

Saba N.F. et al. [143] investigated the efficacy of combination therapy using pembrolizumab (a PD-1 inhibitor) and cabozantinib (a tyrosine kinase inhibitor) in patients with HNSCC. In their study, they observed a treatment response in 52% of patients, disease stabilization in 39%, and disease progression in 9%. Interestingly, the response was observed in both HPV-negative and HPV-positive HNSCC tumors. The researchers concluded that the therapy was well-tolerated and provides a solid foundation for further studies [143].

## 8. Conclusions

Cisplatin remains the gold standard in HNSCC chemotherapy, significantly improving overall survival for patients with this type of cancer. Its effectiveness has been confirmed in numerous clinical trials. However, the use of this chemotherapy agent is not free from side effects. Adverse effects associated with cisplatin include nephrotoxicity, neurotoxicity, and ototoxicity, which significantly hinder its use in some patients with comorbidities and in the elderly.

In patients who do not tolerate cisplatin well, given its toxic effects, another platinum-based drug, carboplatin, is much better. It exhibits fewer neurotoxic and nephrotoxic effects. Due to its lower toxicity, carboplatin is used to treat patients for whom cisplatin is contraindicated due to poor tolerance. Despite its improved safety profile, carboplatin maintains good therapeutic effectiveness and improves patients’ quality of life. Another drug used in chemotherapy is 5-FU, most often used in combination therapies, yielding beneficial results in improving the well-being of patients with HNSCC. On the other hand, 5-FU exhibits gastrointestinal and cardiovascular toxicity, and its administration is problematic due to the need for long infusions.

Despite the long-term use of these chemotherapeutic agents, there is a lack of knowledge about their long-term side effects, for example, when combined with immunotherapy or targeted therapy. Regarding the information provided regarding carboplatin, knowledge about its effects on cancers with various HPV statuses is limited and requires further investigation. It is also worth noting the insufficient data regarding the analysis of long-term side effects of chemotherapy and its combination therapies. Drug resistance of HNSCC tumors to chemotherapeutic agents is also a significant problem. Combination therapies with targeted therapies and nanoparticles, which increase the specificity of drug action, can help achieve this.

Recently, immunotherapy has proven to be a breakthrough in HNSCC cancer treatment. This approach does not typically involve direct action on cancer cells. The essence of this therapy is to regulate the immune system’s response using ICIs (PD1 and PD-L1), which positively impact survival compared to platinum-based chemotherapy agents. Furthermore, PD-1 and PD-L1 inhibitors have been used not only in HNSCC but also in other types of cancer, such as melanoma, lung, pancreatic, colon, and prostate cancer. The cited clinical trials (KEYNOTE-012, -055, -B10) also indicate that pembrolizumab is effective both as monotherapy and in combination with chemotherapy agents. Pembrolizumab monotherapy may be a good alternative for patients who do not tolerate chemotherapy well. However, immunotherapy has several limitations. The first is that patient response to this type of treatment is not universal. Patients with HNSCC rarely demonstrate a complete response to treatment, and up to 60% of patients do not respond to immunotherapeutic agents. Tregs, present in the tumor microenvironment, significantly limit the effectiveness of immunotherapy. Tregs have been shown to support immunosuppressive activity, aid in immune evasion, and be associated with a poorer prognosis.

Despite the limitations of immunotherapy, the use of this approach in the perioperative setting may show promise. The results indicate that administering a single dose of pembrolizumab before surgery may improve tumor response and reduce the risk of relapse, which deserves further investigation. Furthermore, immunotherapy combined with standard treatment approaches may prove an interesting avenue for future research. Further information is available here. Based on the efficacy data of immunotherapy, prior testing of the patient’s PD-L1 expression level may be helpful in maximizing treatment effectiveness. Another interesting avenue for future research may be regulating Treg function in HNSCC tumors to overcome their resistance to immunotherapy.

A leading group of drugs identified in our publication was CDK inhibitors. Third-generation CDK inhibitors (palbociclib, ribociclib, abemaciclib) were indicated as promising tools in the fight against HNSCC, especially the non-HPV subtype. It has been observed that the majority of HNSCC patients exhibit aberrations in the CDKN2A and CCND1 genes, which may be a rationale for using CDK4/6 inhibitors as targeted therapy. It has also been observed that the use of CDK4/6 inhibitors in combination with cetuximab or chemotherapy demonstrates synergistic effects and greater clinical potential. However, monotherapy with this type of drug has proven to be limited. One of the third-generation drugs, palbociclib, demonstrated tumorigenic activity in Rb+ cell lines but was ineffective in the Rb- phenotype, demonstrating the important role of Rb protein status and its potential use as a marker of treatment response.

Despite their potential in the treatment of HNSCC, CDK inhibitors are subject to limitations, including insufficient selectivity and clinical efficacy, along with demonstrated toxicity and a lack of predictable benefits. Furthermore, not all aberrations in CDK2A and CCND1 translate into actual activation of the CDK4/6 pathway, which complicates patient selection. Furthermore, Phase II clinical trials using palbociclib in combination with cetuxamib failed to demonstrate significant therapeutic effects, suggesting limited efficacy of this combination in certain populations. A significant limitation of CDK inhibitors is their hematological and gastrointestinal toxicity, which can complicate long-term use of this treatment strategy. Future studies on CDK inhibitors should focus on identifying predictive biomarkers, such as Rb protein phosphorylation status, which will allow for a more effective selection of patients eligible for this type of therapy. Investigating CDK inhibitors also requires understanding the role of these inhibitors in non-HPV-related HNSCC, where the regulation of cyclin D1/CDK4/6 plays a significant role. Interesting avenues for future research on CDK inhibitors may also include their use in combination therapies with chemotherapy, immunotherapy, or radiotherapy, which could increase the efficacy of CDK inhibitors and reduce the chemoresistance of HNSCC tumors.

Another strategy proposed for the treatment of HNSCC is the inhibition of PARP1, which plays a key role in the cellular response to DNA damage and replication stress, including through protein paralysis, replication fork stabilization, and interaction with p53. HNSCC cells demonstrate increased PARP1 expression, particularly in invasive margins and precancerous lesions, suggesting that PARP1 may be important for tumor progression and invasion. Studies have shown that PARP1 inhibition (using olaparib) demonstrates promising antitumor activity in HNSCC cells, particularly in conditions of HR deficiency, radiation-induced stress, and in combination with cetuximab or KDM inhibitors. Olaparib monotherapy has limited efficacy, but in combination with other therapies—radiotherapy, cetuximab, cisplatin, or KDM inhibitors—it demonstrates a synergistic effect, leading to increased apoptosis, increased p21 expression, and DNA damage, regardless of HPV status. Furthermore, imaging with ^18^F-PARPi targeting PARP1/2 appears safe and may be a useful tool for imaging HNSCC lesions. It is worth mentioning that the use of PARP1 inhibitors is associated with limitations. The first is that mutations in HR pathway genes are rare, limiting the number of patients sensitive to PARP monotherapy. The uneven response of cells to PARP inhibitors is also interesting, suggesting the presence of other unidentified resistance mechanisms. However, regarding PET imaging using ^18^F-PARPi, the strategy is not sufficiently well-studied to identify limitations in the use of this approach.

Future directions of research on PARP inhibitors may include investigating the precise mechanisms of resistance in HNSCC cells, particularly those exhibiting increased PARP1 expression. It is also worthwhile to expand studies on combination therapies with cisplatin, cetuximab, radiotherapy, or KDM inhibitors to select drug doses and enhance their therapeutic efficacy. Future research should certainly include the clinical development of PARP1/2-targeted imaging to determine its role in HNSCC diagnosis and determine the long- and short-term effects of its use. In PARP inhibitor trials, it is also necessary to identify subgroups of patients who would benefit most from this strategy, taking into account HPV status and the nature of the cancer.

Biomarkers such as HPV DNA/mRNA, HPV-E6, EBV RNA, p16^Ink4a, and the more recently identified NNMT and PON2 play critical roles in the diagnosis, prognosis, and prediction of treatment response in head and neck squamous cell carcinoma (HNSCC). Increasing attention is being directed toward biomarkers with both prognostic and predictive utility, enabling personalized therapeutic strategies.

NNMT and PON2 have emerged as particularly promising candidates. NNMT expression varies with cancer type and stage and may reflect tumor cell differentiation. Its differential expression between tumor and adjacent normal tissue has been associated with metastatic potential and treatment response. PON2, through its anti-apoptotic and antioxidant activity, appears to support tumor cell survival and resistance to chemotherapy. Both enzymes are under investigation as therapeutic targets. While NNMT inhibitors are in development, challenges remain concerning their bioavailability and in vivo efficacy. PON2 silencing has shown potential to enhance chemosensitivity, warranting further mechanistic exploration. Future studies should focus on optimizing NNMT-targeting compounds, elucidating PON2’s role in therapy resistance, and improving diagnostic precision. However, limitations such as variable expression, limited sensitivity and specificity, and interpatient variability must be addressed before these markers can be adopted in routine clinical practice. Despite early-stage evidence, their full therapeutic and diagnostic potential requires further validation in clinical settings.

## Figures and Tables

**Table 3:** Current trends in targeted therapy research for HNSCC—summary of clinical trials from the last five years, https://clinicaltrials.gov (accessed on 26 June 2025).

Phase	Aim of Study	Molecular Target	Status	Results	No. of Research
I/II	To assess the safety, determine the recommended combination dosing, and evaluate the early antitumor activity of tipifarnib and alpelisib in patients with HRAS overexpression and/or PIK3CA mutation and/or amplified recurrent/metastatic head and neck squamous cell carcinoma.	PI3K inhibition	Active, not recruiting	No results published yet	NCT04997902
I	To evaluate the safety and tolerability of a conventional PI3K-alpha inhibitor (TOS-358) in adult patients with solid tumors, breast cancer, HNSCC, urothelial cancer, and endometrial cancer; monotherapy.	PI3K inhibition	Recruiting	No results published yet	NCT04997902
Ib/II	To assess the safety, tolerability, and preliminary efficacy of duvelisib in combination with pembrolizumab in participants with R/M HNSCC.	PI3K inhibition	Terminated	Study stopped by a sponsor. Due to small number of participants (2 patients receiving treatment), no data published	NCT04193293
II	Evaluation of the efficacy and safety of preoperative administration of MRG003 in combination with pucotenlimab ± cisplatin injection.	Anti-EGFR	Active, not recruiting	Completed	NCT06530914
-	Evaluation of EGFR expression in OSCC patients.	EGFR level detection	Completed	p16, p53, and EGFR were positive in 60%, 44%, and 58% of cases, respectively. Significant associations were observed between p16 and age, tumor location, abnormal sexual habits, and lymph node involvement. p53 expression correlated with age and sexual habits, and p16 expression significantly co-occurred with p53 and EGFR	NCT06606301
I/II	To assess the safety, tolerability, pharmacokinetics, and antitumor activity of YH32364 in patients with locally advanced or metastatic solid tumors.	Bispecific antibody targeting EGFR and 4-1BB	Recruiting	No results published yet	NCT06975410
II	To assess the safety and feasibility of neoadjuvant PD-1 blockade, alone or in combination with TPF chemotherapy, in patients with locally advanced, resectable oral squamous cell carcinoma.	Inhibition of the immune checkpoint PD-1	Completed	No results published yet	NCT04649476
Ib/IIa	Evaluation of the efficacy, safety, and pharmacodynamics of CyPep-1 administered intralesional in combination with pembrolizumab (anti-PD-1). Analysis of the antitumor activity and local and systemic immunological effects of CyPep-1 on lesions after and without injection.	Inhibition of the immune checkpoint PD-1 and the use of a tumor membrane-targeting protein	Completed	No results published yet	NCT05383170
I	The aim of the study is to assess safety, tolerability, and dose-limiting toxicity, and to determine the maximum tolerated and recommended Phase 2 dose for the further development of PRT3645.	CDK 4/6 inhibition	Completed	No results published yet	NCT05538572
II	The aim of the study is to evaluate new combination therapies targeting the EGFR pathway and CDK4/6 inhibitors in patients with PD-1-resistant head and neck squamous cell carcinoma.	CDK4/6 inhibition and EGFR inhibition	Recruiting	No results published yet	NCT05721443
II	The aim of the study is to evaluate new combination therapies targeting the EGFR pathway and CDK4/6 inhibitors in patients with PD-1-resistant head and neck squamous cell carcinoma.	CDK4/6 inhibition and PD-1 inhibition	Active, not recruiting	No results published yet	NCT06199271
I/II	The aim of the study is to evaluate [18F]-olaparib as a radioactive tracer for imaging PARP expression in tumors using PET.	PARP inhibition	Active, not recruiting	No results published yet	NCT06482307
II	Evaluation of the effectiveness of maintenance therapy with Dostarlimab and Niraparib in patients with surgically removed HNSCC.	PD-1 and PARP inhibition	Recruiting	No results published yet	NCT04681469

CDK4/6—cyclin-dependent kinase 4 and 6; CyPep-1—Cytovation Peptide 1; EGFR—epidermal growth factor receptor; HNSCC—head and neck squamous cell carcinoma; HRAS—HRas Proto-Oncogene; OSCC—oral squamous cell carcinoma; PARP—poly (ADP-ribose) polymerase; PD-1—death protein; PI3K—phosphoinositide 3-kinase; PRT3645-next-generation, PIK3CAPhosphatidylinositol-4,5-Bisphosphate 3-Kinase Catalytic Subunit Alpha.

## Data Availability

Available on request and with regulations.
